# Supplementary material for: Detecting protein and DNA/RNA structures in cryo-EM maps of intermediate resolution using deep learning
Source: Nat Commun. 2021 Apr 16;12:2302. doi: 10.1038/s41467-021-22577-3 (PMC8052361; doi:10.1038/s41467-021-22577-3)
Supplement: Supplementary file 1 — Supplementary Information [file 41467_2021_22577_MOESM1_ESM.pdf]

Supplementary Information for

Detecting Protein and DNA/RNA Structures in Cryo-EM Maps of Intermediate Resolution Using Deep Learning

Xiao Wang[1], Eman Alnabati[1], Tunde W Aderinwale[1], Sai Raghavendra Maddhuri Venkata Subramaniya[1], Genki Terashi[2], and Daisuke Kihara[2, 1, *]

[1]Department of Computer Science, Purdue University, West Lafayette, IN, 47907, USA, [2]Department of Biological Sciences, Purdue University, West Lafayette, IN, 47907, USA.

* Contact: dkihara@purdue.edu

Supplementary Table 1. The dataset of experimental maps.

Fold	Representative entries	Entries in the same clusters as representatives
**1**	EMD-7845 (6DBL), EMD-8518 (5U8S), EMD-6906 (5ZAM), EMD-7290 (6BU9), EMD-4671 (6QY3)	EMD-4670 (6QXT)
**2**	EMD-9316 (6N1P), EMD-4141 (5M1S), EMD-3545 (5MQF), EMD-0244 (6HMS), EMD-4139 (5M0R)	EMD-3198 (5FKV), EMD-3202 (5FKW), EMD-3683 (5NRL), EMD-6889 (5Z56), EMD-6890 (5Z57), EMD-9621 (6AH0)
**3**	EMD-6803 (5Y3R), EMD-4242 (6FEC), EMD-2810 (4D5Y), EMD-7103 (6BJS)	EMD-5942 (3J6X), EMD-5943 (3J6Y), EMD-5976 (3J77), EMD-5977 (3J78), EMD-2683 (4UJC), EMD-2682 (4UJD), EMD-2620 (4UJE), EMD-5036 (4V69), EMD-1849 (4V6K), EMD-5775 (4V7B), EMD-5799 (4V7C), EMD-5800 (4V7D), EMD-8190 (5K0Y), EMD-4078 (5LMS), EMD-4122 (5LZB), EMD-3581 (5MYJ), EMD-3661 (5NO2), EMD-3662 (5NO3), EMD-3663 (5NO4), EMD-8621 (5UZ4), EMD-7014 (6AWB), EMD-7015 (6AWC), EMD-7016 (6AWD), EMD-3637 (6FXC), EMD-0057 (6GSM), EMD-0058 (6GSN), EMD-0139 (6H58), EMD-0195 (6HCM), EMD-0197 (6HCQ), EMD-4427 (6I7O), EMD-2813 (4D67), EMD-3561 (5MS0), EMD-3696 (5NSS), EMD-7439 (6CA0), EMD-3580 (5MY1)
**4**	EMD-2784 (4V1M), EMD-3949 (6ESH), EMD-4075 (5LMP), EMD-3305 (5FUR), EMD-8131 (5IVW)	EMD-2785 (4V1N), EMD-2786 (4V1O), EMD-3383 (5FZ5), EMD-8132 (5IY7), EMD-8133 (5IY8), EMD-8134 (5IY9), EMD-8135 (5IYA), EMD-8735 (5VVR), EMD-8737 (5VVS), EMD-6980 (6A5L), EMD-6981 (6A5O), EMD-6982 (6A5P), EMD-6983 (6A5R), EMD-6984 (6A5T), EMD-6985 (6A5U), EMD-4182 (6F42), EMD-6986 (6INQ), EMD-0671 (6J4W), EMD-3948 (6ESG), EMD-3950 (6ESI), EMD-4336 (6G0L), EMD-9843 (6JM9), EMD-0090 (6GYK)

EMDB ID and PDB ID are paired. Representative entries that were used as a test set in the four-fold cross-validation were shown in the Representative column. The list on the right column is entries that are similar (at least one chain in the complex has not less than 25% sequence identity to a chain in a representative entry) to one of the representative entries. Entries in the similar clusters were used for training when a different fold was used for testing. See Methods for more details of the dataset construction.

Supplementary Fig. 1

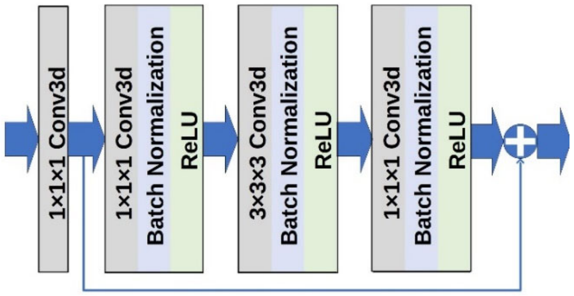

The architecture of Residual block. 6 blocks are used in the phase 1 network (Figure 1). A Residual block consists of a combination of convolutional layer, batch normalization layer, and ReLU activation with residual connections to avoid gradient collapse and previous layer's information loss.

Supplementary Fig. 2.

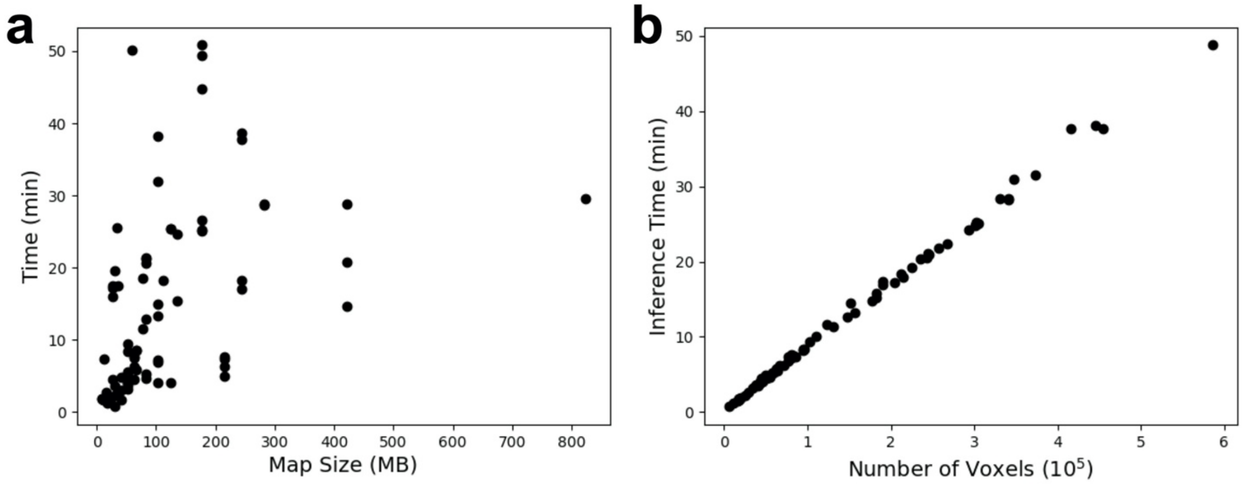

The computational time of inference on 84 experimental maps. **a,** Time spent for processing maps with a full procedure of the following four steps. The x-axis shows the map file size in mega bytes (MB):

1) Interpolating the original map with a grid size of 1.0 Å (using the process_map.Reform_Map_Voxel.Reform_Map_Voxel function provided in the Emap2sec+ GitHub repository); 2) Apply a contour level to the map and prepare voxel input data (running the process_map.Build_Map program); 3) Time for Emap2sec+ to make the phase 1 structure detection; 4) Time for Emap2sec+ to make the phase 2 detection. In this plot we excluded EMD-7304, which costed an exceptionally long time of 929 minutes. This map has a grid size of 0.76 Å, which is smaller than 1 Å, and needed to use a different slower function, process_map.Reform_Map_Voxel.Reform_Map_Voxel_Final. to adjust the grid size in step 1. This took 917 minutes. The speed of this function is much slower due to a different library used. Thus, here 83 maps were plotted. **b**, the relationship of number of voxels and the time for inference (phase1+phase2). The computational times of the maps were measured with the use of NVIDIA GTX 2080 GPU.

Supplementary Table 2. GPU memory usage in inference.

Batch Size	32	64	128	256
GPU Memory (GB)	1.4	2.1	3.2	6.0

Memory usage was measured on NVIDIA GTX 2080 GPU with a batch size 256, 128, 64, and 32 in inference. The batch size determines the number of voxels to process in parallel. The memory usage does not depend on the size of the input map as input data to Emap2sec+ is voxels.

User may choose a small batch size to allow Emap2sec+ run on their local machine with a GPU with a small memory, which will not influence the accuracy but may increase the computation time. The default batch size is 256 in our Emap2sec+. The batch size can be set by a parameter --batch_size in the Emap2sec+ script.

Supplementary Table 3. Summary of the structure detection for simulated maps.

Measure	Resolution	α helices	β strands	Others	DNA/RNA	All
**Voxel-based F1 score**	6 Å	0.835 (0.868)	0.706 (0.762)	0.723 (0.775)	0.902 (0.907)	0.844 (0.875)
	6 -10 Å	0.797 (0.828)	0.640 (0.698)	0.669 (0.719)	0.807 (0.811)	0.798 (0.829)
	10 Å	0.771 (0.803)	0.602 (0.665)	0.634 (0.689)	0.797 (0.801)	0.776 (0.809)
**Voxel-based Accuracy**	6 Å	0.830 (0.857)	0.771 (0.816)	0.705 (0.765)	0.907 (0.912)	0.845 (0.876)
	6-10 Å	0.807 (0.829)	0.705 (0.753)	0.650 (0.706)	0.814 (0.817)	0.799 (0.829)
	10 Å	0.773 (0.796)	0.701 (0.755)	0.598 (0.662)	0.805 (0.810)	0.778 (0.810)
**Residue Q4**	6 Å	0.863 (0.869)	0.816 (0.833)	0.738 (0.767)	0.940 (0.944)	0.849 (0.864)
	6-10 Å	0.838 (0.846)	0.747 (0.775)	0.677 (0.704)	0.839 (0.840)	0.804 (0.821)
	10 Å	0.807 (0.820)	0.751 (0.783)	0.625 (0.666)	0.829 (0.836)	0.778 (0.802)
**Segments**	6 Å	0.947 (0.950)	0.935 (0.940)	-	-	-
	6-10 Å	0.933 (0.944)	0.864 (0.864)	-	-	-
	10 Å	0.904 (0.926)	0.835 (0.892)	-	-	-

In parentheses for different evaluation metrics, we used relaxed measure to calculate, where a cube with multiple labels will be considered as correct if model predicts one of the multiple labels. The 6-10 Å dataset contains simulated maps computed for a randomly selected resolution between 6 to 10 Å. Similar to the 6 Å and 10 Å datasets, networks for 6-10 Å were trained specifically for this dataset.

The residue Q4 is the residue/nucleoside level accuracy (recall). The segment-based accuracy considers the fraction of segments that are successfully identified. More detailed definitions are included in the Methods section.

Supplementary Fig. 3.

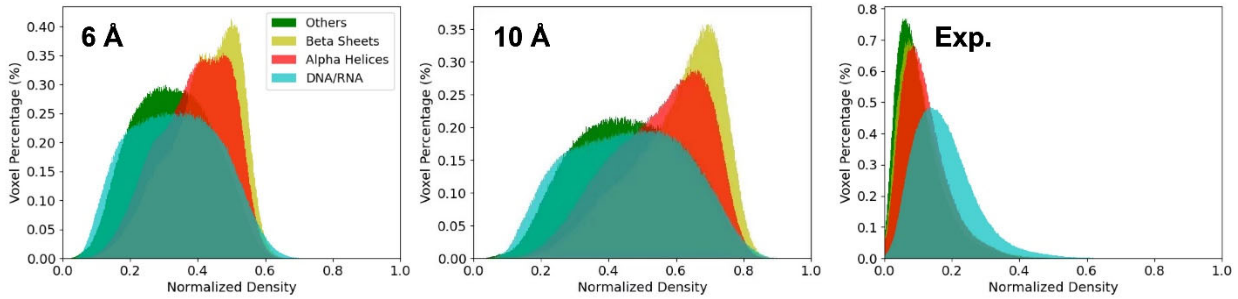

Distribution of normalized densities of four structural classes.

The distributions of the 108 test maps simulated at 6 Å, 10 Å as well as 84 experimental maps are shown.

Supplementary Fig. 4. Structure detection of lob B of the TFIID-IIA and promoter DNA complex using the 4.5 Å resolution map (EMD-9298).

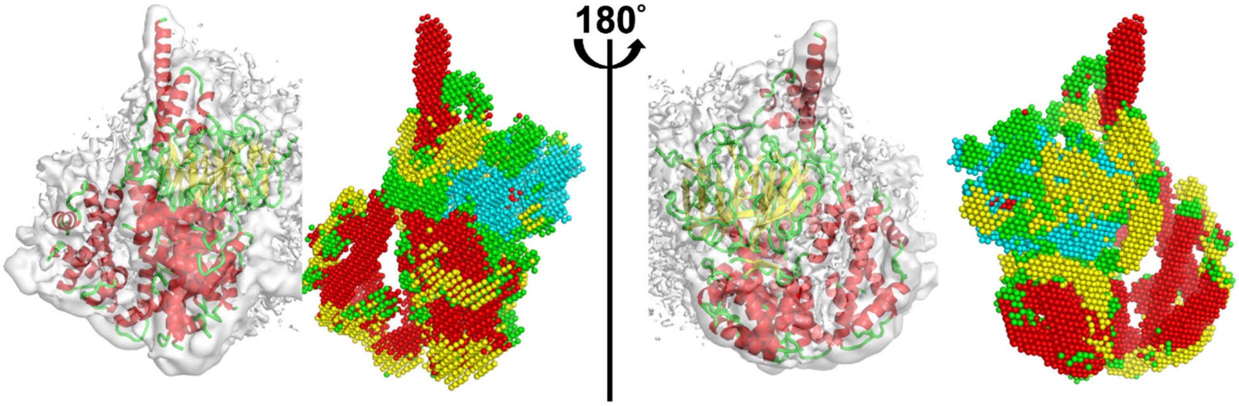

Structures in lobe B was detected in EDM-9298, determined at 4.5 Å resolution. Detected structures were compared with corresponding region, 953 amino acids, in 6MZC. We used a contour level of 0.01. Voxel-based F1 score: 0.574; Voxel-based accuracy: 0.509; Q3(Q4): 0.517. Results for other metrics are listed in the bottom of Supplementary Data 2.

Supplementary Fig. 5. Structure detection results by Haruspex on the experimental map datasets.

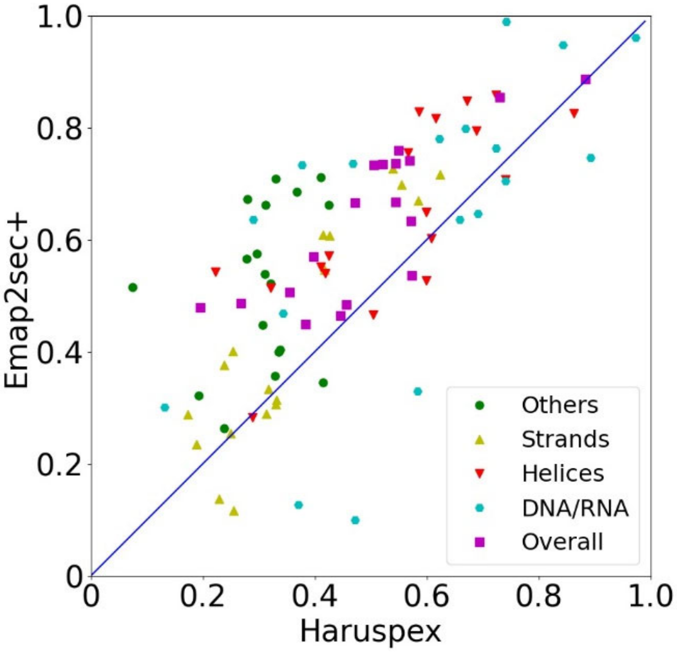

We ran Haruspex (Mostosi et al., Angew Chem Int Ed Engl, 2020) the experimental map datasets and compared the voxel-based F1 score with Emap2sec+. We used the voxel-based metric in this comparison because Harruspex was evaluated at voxel level in their paper.
