# Supplementary material for: Detecting protein and DNA/RNA structures in cryo-EM maps of intermediate resolution using deep learning
Source: Nat Commun. 2021 Apr 16;12:2302. doi: 10.1038/s41467-021-22577-3 (PMC8052361; doi:10.1038/s41467-021-22577-3)
Supplement: Supplementary file 3 — Description of Additional Supplementary Files [file 41467_2021_22577_MOESM3_ESM.pdf]

Description of Additional Supplementary Files

File Name: Supplementary Data 1
Description: **Structure assignment results for individual maps in the simulated map test set** (in a separate Excel file).
Data are shown for phase 1 and 2 results for simulated maps at 6 Å, 10 Å, and at a resolution randomly selected between 6 to 10 Å. -1 in the table indicates that the structural class does not exist in the protein complex.
To report the phase 1 accuracy, for each voxel that received structure assignment we first normalized the individual probability values from the four binary classification models of ⍰ helix, β strand, other structures, and DNA/RNA, so that they sum up to 1.0, then averaged the values with the probability values of the four classes from the multi-class model.

File Name: Supplementary Data 2
Description: **Structure assignment results for individual maps in the experimental map test set.** (in a separate Excel file).
Data are shown for phase 1 and 2 results for individual experimental maps in the first and the second page. Fold information corresponds to the four-fold grouping of maps shown in Supplementary Table 1. The third page, “Binary evaluation” provides results of classification between proteins and DNA/RNAs. In the fourth and fifth page (phase1_nocontour and phase2_nocontour), results are shown for maps that are not truncated with the author-recommended contour level.

File Name: Supplementary Data 3
Description: **Structure assignment results for individual experimental maps in the test set using only the phase 1 multi-class model.** (in a separate Excel file).
Data are shown for phase 1 results for individual experimental maps using only the multi-class model.
