# Supplementary material for: Detecting protein and DNA/RNA structures in cryo-EM maps of intermediate resolution using deep learning
Source: Nat Commun. 2021 Apr 16;12:2302. doi: 10.1038/s41467-021-22577-3 (PMC8052361; doi:10.1038/s41467-021-22577-3)
Supplement: Supplementary file 7 — Reporting Summary [file 41467_2021_22577_MOESM7_ESM.pdf]

nature research

Corresponding author(s): Daisuke Kihara

Last updated by author(s): 2021-3-12

Reporting Summary

Nature Research wishes to improve the reproducibility of the work that we publish. This form provides structure for consistency and transparency in reporting. For further information on Nature Research policies, see our Editorial Policies and the Editorial Policy Checklist.

Statistics

For all statistical analyses, confirm that the following items are present in the figure legend, table legend, main text, or Methods section.

n/a	Confirmed	
☐	☒	The exact sample size (*n*) for each experimental group/condition, given as a discrete number and unit of measurement
☐	☒	A statement on whether measurements were taken from distinct samples or whether the same sample was measured repeatedly
☒	☐	The statistical test(s) used AND whether they are one- or two-sided<br>*Only common tests should be described solely by name; describe more complex techniques in the Methods section.*
☒	☐	A description of all covariates tested
☒	☐	A description of any assumptions or corrections, such as tests of normality and adjustment for multiple comparisons
☒	☐	A full description of the statistical parameters including central tendency (e.g. means) or other basic estimates (e.g. regression coefficient) AND variation (e.g. standard deviation) or associated estimates of uncertainty (e.g. confidence intervals)
☒	☐	For null hypothesis testing, the test statistic (e.g. *F*, *t*, *r*) with confidence intervals, effect sizes, degrees of freedom and *P* value noted<br>*Give P values as exact values whenever suitable.*
☒	☐	For Bayesian analysis, information on the choice of priors and Markov chain Monte Carlo settings
☒	☐	For hierarchical and complex designs, identification of the appropriate level for tests and full reporting of outcomes
☒	☐	Estimates of effect sizes (e.g. Cohen's *d*, Pearson's *r*), indicating how they were calculated

Our web collection on statistics for biologists contains articles on many of the points above.

Software and code

Policy information about availability of computer code

Data collection	we made the source codes available at GitHub, https://github.com/kiharalab/Emap2secplus SITUS version 3.12 (https://situs.biomachina.org/)
Data analysis	The source code is on Github https://github.com/kiharalab/Emap2secplus We used Pymol (ver2.4) https://pymol.org/2/ and Chimera ver. 1.14 https://www.cgl.ucsf.edu/chimera/ for visualization.

For manuscripts utilizing custom algorithms or software that are central to the research but not yet described in published literature, software must be made available to editors and reviewers. We strongly encourage code deposition in a community repository (e.g. GitHub). See the Nature Research guidelines for submitting code & software for further information.

Data

Policy information about availability of data

All manuscripts must include a data availability statement. This statement should provide the following information, where applicable:

- Accession codes, unique identifiers, or web links for publicly available datasets
- A list of figures that have associated raw data
- A description of any restrictions on data availability

The raw data of the structure models built by our method are provided in Supplementary Information, Supp. Table 1 and Data 1. The simulated EM map dataset can be downloaded from https://doi.org/10.5281/zenodo.4601546. The experimental EM maps can be downloaded from EMDB (https://www.emdataresource.org/).

Field-specific reporting

Please select the one below that is the best fit for your research. If you are not sure, read the appropriate sections before making your selection.

☒ Life sciences ☐ Behavioural & social sciences ☐ Ecological, evolutionary & environmental sciences

For a reference copy of the document with all sections, see nature.com/documents/nr-reporting-summary-flat.pdf

Life sciences study design

All studies must disclose on these points even when the disclosure is negative.

Sample size	We used EM maps that are either simulated from atomic detailed structures (108maps) from PDB and 83 experimental EM maps downloaded from EMDB. We tried our best to collect as much as we can from EMDB. These were all the EMDB entries that satisfied following criteria: 1) they are non-redundant; 2) have associated structure files in PDB; 3) have both proteins and DNA/RNA. For more details please see the Methods section.
Data exclusions	The data selection steps are described in Methods. No data exclusions are carried here.
Replication	We performed cross-validation to make sure that training dataset of the deep learning models and testing set are different. All the codes and data are also released and user can verify our results by running our code on our data.
Randomization	randomization is not applicable because we used all the EMDB entries which satisfied above mentioned criteria that were available at that time of the experiment.
Blinding	Blinding is not relevant to this study because we collected randomization is not applicable because we used all the EMDB entries which satisfied above mentioned criteria that were available at that time of the experiment.

Reporting for specific materials, systems and methods

We require information from authors about some types of materials, experimental systems and methods used in many studies. Here, indicate whether each material, system or method listed is relevant to your study. If you are not sure if a list item applies to your research, read the appropriate section before selecting a response.

Materials & experimental systems

n/a	Involved in the study	
☒	☐	Antibodies
☒	☐	Eukaryotic cell lines
☒	☐	Palaeontology and archaeology
☒	☐	Animals and other organisms
☒	☐	Human research participants
☒	☐	Clinical data
☒	☐	Dual use research of concern

Methods

n/a	Involved in the study	
☒	☐	ChIP-seq
☒	☐	Flow cytometry
☒	☐	MRI-based neuroimaging
